# Integrated health service delivery networks: concepts, policy options and road map for implementation in the Americas

**Published:** 2013-09-25

**Authors:** Lourdes Ferrer

**Affiliations:** International Foundation for Integrated Care, UK

Pan American Health Organization (PAHO)/World Health Organisation introduce its book as a key step to support their member states’ commitment towards the development of Integrated Health Service Delivery Networks, as a preferred modality for health service delivery and especially as a main operational strategy of the Primary Health Care approach.

The book is proposed as a guiding tool to analyse problems related to health services fragmentation. It seeks to support the analysis of the state of a country's Integrated Health Service Delivery Networks in terms of a list of attributes. Policy instruments and institutional mechanisms that can be used to support the strengthening of the network per attribute are then presented. The prioritisation of certain attributes and the choice of instruments and mechanisms should become the backbone of national plans.

The book is organised into five brief chapters. Chapter one guides the understanding of the benefits of Integrated Health Service Delivery Networks for the current health challenges. They expose fragmentation as a main challenge in Latin America, due to a historical development of subsystems that were ‘targeted to specific strata of the populations’ (for example, type of employment, income, urban or rural residence), and to vertical programmes sponsored by international health initiatives.

Chapter two presents the concept of ‘comprehensive, integrated and continuous health services” and of Integrated Health Service Delivery Networks, its different modalities and benefits. Integrated Health Service Delivery Networks or Organized Health Services Systems, or Clinically Integrated Systems, or Integrated Health Organizations, are defined as ‘a network of organizations that provides, or makes arrangements to provide, equitable, comprehensive, integrated, and continuous health services to a defined population and is willing to be held accountable for its clinical and economic outcomes and the health status of the population served’.

Fourteen essential attributes of Integrated Health Service Delivery Networks grouped in four principal domains are proposed as a flexible template to assess a country's situation, propose priority interventions and assess progress in chapter three. They are distributed in four domains: governance, organization and management, model of care and financial incentives (Figure 1).

In chapter four, public instruments and institutional mechanisms that support the development of Integrated Health Service Delivery Networks are proposed. Their relevance in 4 different settings was analysed. Those settings were (1) highly segmented health systems; (2) systems without universal coverage and access; (3) systems with decentralisation that fragments service delivery; and (4) systems with separation of functions and multiple service providers.

The final chapter briefly presents lessons learned from the past successful developments and the two phases of the initiative. The first phase (2009–2010) is about analysis of the country's situation in terms of the attributes and the development of a national plan. The second phase (from 2011) involves implementation of the national plan and ongoing evaluation. For more information see the next publication of the initiative [1].

The book is very well structured and easy to read. It does propose a straightforward tool to discuss a country's situation with respect to the attributes. It also suggests legal, political instruments and clinical and non-clinical interventions that can be used to plan how to progress in each attribute. Unfortunately and already mentioned by the authors, the book does not incorporate the extended analysis of micro- and meso-level mechanisms, and their interactions. More practical examples of the assessment of each attribute in different contexts would have been useful to support professionals wishing to apply the tool.

In conclusion I highly recommend this book to all professionals hoping to support the flourishing of integrated care, especially for the development of macro-policies that support the flourishing of integrated care; for the understanding of the interrelations of different entry points when seeking to develop health care reforms; and for its explicit notion of continuous development and adaptation to changing priorities.

The book has added value in suggesting tools to understand, propose and assess the developmental path towards integrated care. For a similar approach please refer to a recent thesis summary published in IJIC [2]. The key lessons to take away are that integrated care needs long-term commitment, complex, adaptive and whole system approaches.

## Figures and Tables

**Figure 1.**
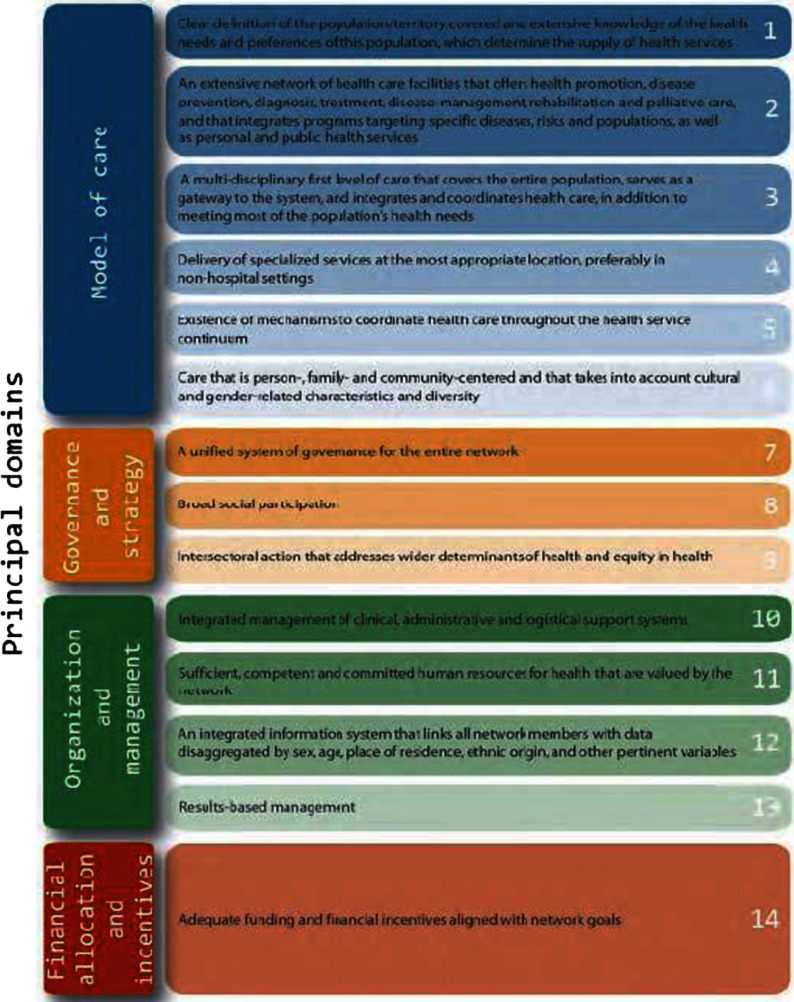
Principal domains.
